# Evaluation of the DLL3-targeting Antibody–Drug Conjugate Rovalpituzumab Tesirine in Preclinical Models of Neuroblastoma

**DOI:** 10.1158/2767-9764.CRC-22-0137

**Published:** 2022-07-11

**Authors:** Kateryna Krytska, Colleen E. Casey, Jennifer Pogoriler, Daniel Martinez, Komal S. Rathi, Alvin Farrel, Esther R. Berko, Matthew Tsang, Renata R. Sano, Nathan Kendsersky, Stephen W. Erickson, Beverly A. Teicher, Kumiko Isse, Laura Saunders, Malcolm A. Smith, John M. Maris, Yael P. Mossé

**Affiliations:** 1 Division of Oncology and Center for Childhood Cancer Research, Children's Hospital of Philadelphia, Philadelphia, Pennsylvania.; 2 Department of Pathology and Laboratory Medicine, Perelman School of Medicine at the University of Pennsylvania, Philadelphia, Pennsylvania.; 3 Department of Biomedical and Health Informatics and Center for Data-Driven Discovery in Biomedicine, Children's Hospital of Philadelphia, Philadelphia, Pennsylvania.; 4 RTI International, Research Triangle Park, North Carolina.; 5 NCI, Bethesda, Maryland.; 6 Abbvie Stemcentrx, South San Francisco, California.; 7 Department of Pediatrics, Perelman School of Medicine at the University of Pennsylvania, Philadelphia, Pennsylvania.

## Abstract

Neuroblastomas have neuroendocrine features and often show similar gene expression patterns to small cell lung cancer including high expression of delta-like ligand 3 (*DLL3*). Here we determine the efficacy of rovalpituzumab tesirine (Rova-T), an antibody–drug conjugated (ADC) with a pyrrolobenzodiazepine dimer toxin targeting DLL3, in preclinical models of human neuroblastoma.

We evaluated DLL3 expression in RNA-sequencing datasets and performed IHC on neuroblastoma patient-derived xenograft (PDX), human neuroblastoma primary tumor and normal childhood tissue microarrays. We then evaluated the activity of Rova-T against 11 neuroblastoma PDX models using varying doses and schedules and compared antitumor activity with expression levels.

DLL3 mRNA was differentially overexpressed in neuroblastoma at comparable levels to small cell lung cancer, as well as Wilms and rhabdoid tumors. DLL3 protein was robustly expressed across the neuroblastoma PDX array, but membranous staining was variable. The human neuroblastoma array, however, showed staining in only 44% of cases, whereas no significant staining was observed in the normal childhood tissue array. Rova-T showed a clear dose–response effect across the 11 models tested, with a single dose inducing a complete or partial response in 3 of 11 and stable disease in another 3 of 11 models. No overt signs of toxicity were observed, and there was no treatment-related mortality. Strong membranous staining was necessary, but not sufficient, for antitumor activity.

Rova-T has activity in a subset of neuroblastoma preclinical models, but heterogeneous expression in these models and the near absence of expression seen in human tumors suggests that any DLL3-targeting clinical trial should be only performed with a robust companion diagnostic to evaluate DLL3 expression for patient selection.

**Significance:**

GD2-directed antibody therapy is standard of care for high-risk neuroblastoma; therapy is toxic, and relapses often occur. DLL3, an inhibitory Notch ligand, is overexpressed in several neuronal cancers. A DLL3-targeting ADC showed objective activity only in neuroblastoma models with high DLL3 expression. These data provide vigilance about clinical development of DLL3 immunotherapies for neuroblastoma.

## Introduction

Neuroblastoma arises from neural crest cells of the developing sympathetic nervous system and accounts for 12% of all childhood cancer mortality (1). This disease remains a significant challenge largely due to its underlying biological heterogeneity, and outcomes for patients with the high-risk form of the disease remain poor despite intensive upfront chemoradioimmunotherapy, with over 50% of patients ultimately dying and survivors burdened with significant treatment-related morbidities (2). Neuroblastoma is the only pediatric solid tumor with an FDA-approved immunotherapy, and three separate mAbs that target GD2 are commercially available. While a randomized phase III study showed a 10% improvement in relapse-free survival, the therapy is toxic, and relapses occur on or after therapy (3, 4). New immunotherapeutic strategies are clearly needed.

Neuroblastomas have neuroendocrine features and often show similar expression patterns to small cell lung cancer (SCLC) as well as other neuroendocrine cancers. Rovalpituzumab tesirine (Rova-T) is an antibody–drug conjugate (ADC)-targeting delta-like protein 3 (DLL3), a Notch ligand that inhibits Notch signaling (5). DLL3 is highly expressed in SCLC and large-cell neuroendocrine carcinoma (LCNEC) models and minimally expressed in normal tissues (6). Rova-T is an ADC comprised of a humanized anti-DLL3 mAb conjugated to a DNA-damaging pyrrolobenzodiazepine (PBD) dimer toxin that has been shown to induce sustained tumor regression across neural crest–derived malignancies including SCLC and LCNEC patient-derived xenograft (PDX) models (5). Noting in our RNA-sequencing data that the median *DLL3* expression level in neuroblastomas was higher than in SCLC, here we sought to develop DLL3 as an immunotherapeutic target for high-risk neuroblastoma.

## Materials and Methods

### Tumor and Normal Tissue Expression Profiling

RNA-sequencing data from patients with high-risk neuroblastoma (*n* = 126), osteosarcoma (*n* = 88), rhabdoid tumor (*n* = 68), and Wilms tumor (*n* = 136) were retrieved from the Therapeutically Applicable Research to Generate Effective Treatments project. RNA-sequencing data for 79 patients with small cell lung cancer were retrieved from the GSE60052 dataset in the NCBI Gene Expression Omnibus database. Lung adenocarcinoma (*n* = 230), lung squamous cell carcinoma (*n* = 501), and mesothelioma (*n* = 87) RNA-sequencing datasets were retrieved from The Cancer Genome Atlas. Normal tissue RNA-sequencing data were obtained by the Genotype-Tissue Expression Project (GTEx). All RNA-sequencing datasets were aligned using STAR and gene-level expression was quantified with RSEM normalization using hg37 as reference genome and Gencode v23 gene annotation. RNA sequencing for the PDX models was performed and analyzed as described previously (7). Fragments Per Kilobase of transcript per Million mapped reads (FPKM) data were available from 21 of 35 neuroblastoma PDX models and were correlated with IHC quantification as described below.

### Tissue Microarray Construction

All tissue microarrays (TMA) were constructed using standard methods (8, 9). Each tumor sample was punched in duplicate using 0.6-mm cores with a Galileo CK3500 Tissue Microarrayer (Integrated System Engineering SRL). For the neuroblastoma PDX array, PDX bearing mice were euthanized when the tumor reached approximately 1 cm^3^ in volume, the tumor was harvested and immediately placed into a 50 mL conical tube with enough 10% neutral buffered formalin (NBF, Thermo Fisher Scientific) to cover the entire tumor (minimum 10:1 formalin to tissue ratio). The tissue was fixed in 10% NBF for 24–48 hours depending on the size of tumor. Fixed tumor tissue was processed in the Excelsior ES tissue processor from Thermo Fisher Scientific using the standard overnight protocol. Formalin-fixed, paraffin-embedded tissue blocks were sectioned, and hematoxylin and eosin (H&E) staining was performed to obtain a template guide slide to match the face of each tissue block. H&E slides were reviewed to validate the sample quality and preservation. PDX samples were excluded if they showed greater than 20% necrosis or inadequate tissue preservation. Slides were annotated to create a template for punching, then used as guides for core selection as described previously (10). The neuroblastoma PDX TMA was constructed of 35 distinct PDX tumor models that were collected in duplicate for a total of 70 tumor samples. Two 0.6-mm cores were punched per each tumor, resulting in a total of 140 tissue sample cores along with nine control tissues (placenta, human brain cortex, human brain cerebellum, human tonsil, human adrenal cortex, human adrenal medulla, murine brain cortex, murine brain cerebellum, and murine adrenals). Human placenta was used as an orientation marker on the array to mark the starting point (upper left-hand corner), and a row down the middle to aid in manual reading of the array.

For the human neuroblastoma tumor and normal pediatric tissue arrays, all samples were collected and deidentified at Children's Hospital of Philadelphia from 2005 to 2016 under Institutional Review Board approval. The normal childhood TMA represents tissues from patients ranging in age between 0 and 253 months (Supplementary Fig. S1). The human neuroblastoma array included 64 tumors from patients between the ages of 0 months to 12 years of age. Eight of these samples were from cases with matched primary and metastatic tumors.

### IHC and Microarray Scoring Methodology

IHC with rabbit anti-DLL3 SP347 (Ventana, 790-7016) and anti-CD56 (Cell Marque, 156-R-95) antibodies was performed on formalin-fixed paraffin-embedded TMA slides. Staining was performed on a Bond Max automated staining system (Leica Biosystems). The Bond Refine polymer staining kit (Leica Biosystems) was used. The standard IHC protocol F was used apart from the mouse polymer step, which was excluded. For CD56 staining, the primary antibody incubation time was extended to 1 hour. DLL3 antibody was prediluted and antigen retrieval was performed with E1 (Leica Biosystems) retrieval solution for 20 minutes. CD56 was used at a 1:200 dilution with E2 (Leica Biosystems, AR9640) retrieval solution for 20 minutes. Slides were rinsed, dehydrated through a series of ascending concentrations of ethanol and xylene, and then covered with coverslips. Stained slides were then digitally scanned at 20× magnification on an Aperio CS-O slide scanner (Leica Biosystems). TMAs were scored for the most prominent intensity (0–3 with 1 representing equivocal, 2 weak, and 3 strong positive staining) as well as for percentage of staining. Both the overall staining (any pattern) and membranous specific staining patterns were recorded. A modified H score was calculated as intensity multiplied by percentage of positively stained cells. All scores for each of the two tumors per PDX model were averaged for the final score.

### Mouse Studies

All xenograft studies were conducted in compliance with protocols approved by the Institutional Animal Care and Use Committee approved by The Children's Hospital of Philadelphia. Felix-PDX, COG-N-452x, COG-N-519x, COG-N-415x, NB-FLY-623m, KWK-6062x, COG-N-421x, and COG-N-424x PDXs, and SH-SY5Y and SK-N-AS cell line xenografts (CDX) were implanted subcutaneously into the right flanks of female CB17 SCID mice (CB17/Icr-Prkdc^scid^/IcrIcoCrl, Charles River Laboratories, strain code 236). When tumors reached 200–300 mm^3^, the animals were randomized into groups of 2–10 mice per arm. Felix-PDX, COG-N-452x, COG-N-519x, or COG-N-415x tumor-bearing mice *n* = 8–10 were enrolled in randomized controlled preclinical trials and dosed via intraperitoneal injection with one single dose according to the following treatments (vehicle, Rova-T at 0.1, 0.3, and 0.6 mg/kg, or IgG1-ADC at 0.6 mg/kg). In a separate study, the COG-N-415x model was treated with 1 or 3 weekly injections of 1 mg/kg of Rova-T, IgG1-ADC, or vehicle. NB-FLY-623m, KWK-6062x, COG-N-421x, COG-N-424x, SH-SY5Y, and SK-N-AS tumor-bearing mice were part of *n* = 2 animal trials and dosed intraperitoneally with one single dose of vehicle or Rova-T at 0.1 and/or 0.6 mg/kg. Tumor volume was estimated using the spheroid formula = (π/6) × (*a* + *b*/2)^3^, where “*a*” and “*b*” are two diameters measured with an electronic caliper. Total body weight and tumor volume were recorded two to three times weekly. Events were defined as quadrupling of a mouse's tumor volume from day zero.

### Statistical Methods

The exact time to event is estimated by interpolating between the measurements directly preceding and following the event, assuming log-linear growth. Differences in event-free survival (EFS) between experimental groups (e.g., treated vs. controls) are tested using the Peto and Peto modification of the Gehan–Wilcoxon test (*α* = 0.05, two-sided alternative) and plotted as Kaplan–Meier EFS curves.

The objective response categories are progressive disease (PD, which is subdivided among treated mice into PD without and with growth delay, PD1 and PD2, respectively), stable disease (SD), partial response (PR), complete response (CR; no measurable tumor mass for one recording), and maintained complete response (no measurable tumor mass for at least three consecutive weekly recordings). Response rate is defined as the percentage of mice with PR or better. Mice experiencing a possible treatment-related death (i.e., drug toxicity), mice with failed engraftment, and mice which unexpectedly die for reasons unrelated to treatment are excluded from statistical analyses of time-to-event, minimum tumor volume, and objective response. For experiments of groups of *n* = 2 mice per group, we did not perform any statistical tests of group differences but have reported all other summary statistics.

All studies performed had written informed consent from the patients when relevant, studies were conducted in accordance with recognized ethical guidelines (including the Declaration of Helsinki, CIOMS, Belmont Report, U.S. Common Rule), and the studies were approved by an Institutional Review Board.

### Data Availability Statement

Data were generated by the authors and included in the article.

## Results

### DLL3 mRNA is Expressed in Neuroblastoma and Other Pediatric Solid Tumors

**FIGURE 1:**
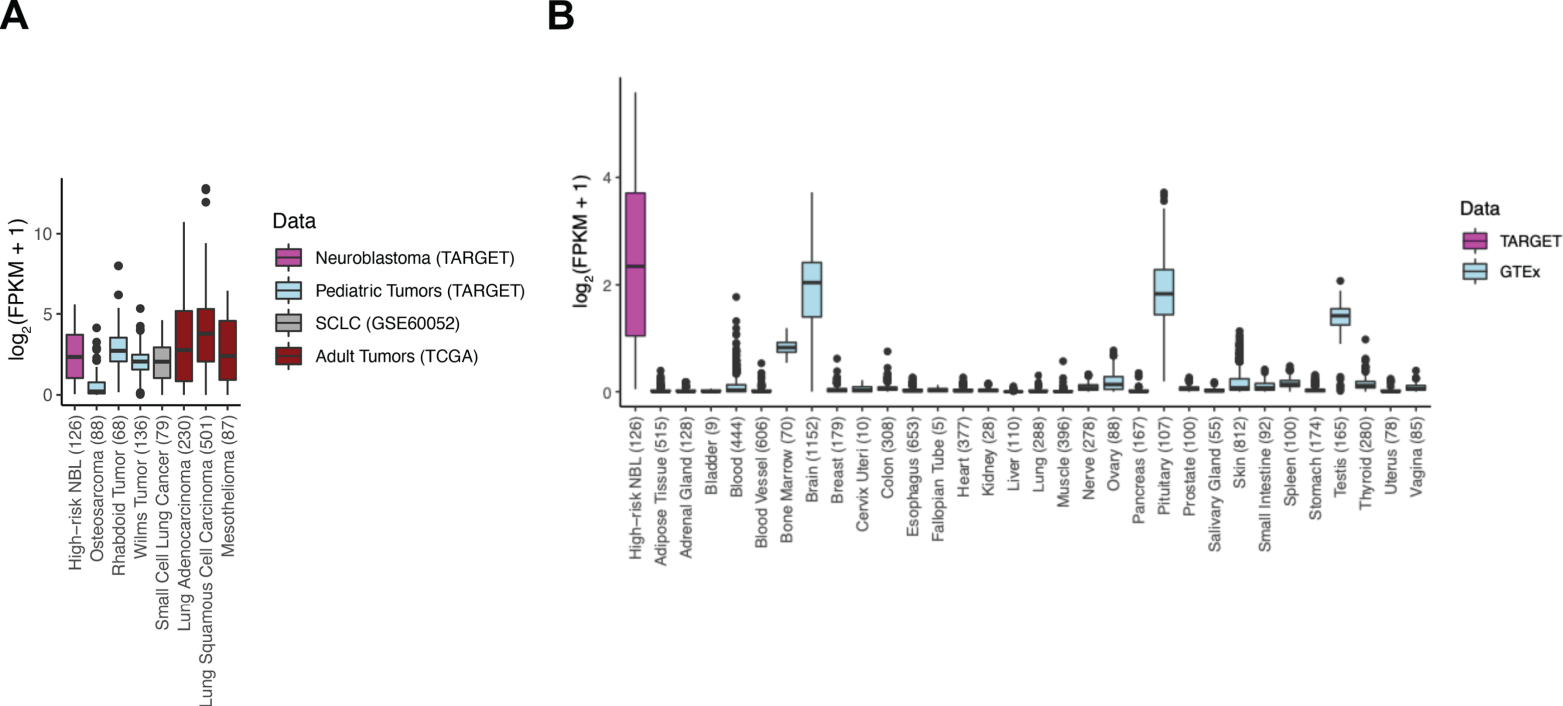
*DLL3* is expressed in neuroblastoma and other pediatric solid tumors. Boxplots displaying *DLL3* expression (log_2_ FPKM) in patients with high-risk neuroblastoma (*n* = 126), SCLC (*n* = 79), and other pediatric solid tumors (*n* = 292; **A**). **B,***DLL3* expression in solid tumors were compared with normal tissue expression data extracted from the GTEx consortium (*n* = 7,859 samples, across 31 unique tissues). The number of samples of each tissue is within the parentheses on the *X*-axis.

We used harmonized RNA-sequencing data to compare DLL3 gene expression of high-risk neuroblastoma primary tumors (*n* = 126) to other pediatric and adult tumors (*n* = 1,189) as well as 7859 GTEx samples across 31 unique normal tissues. In comparison with normal tissues, except for brain, pituitary gland, and testes, *DLL3* showed significantly higher expression in neuroblastoma and some other pediatric solid tumors, such as malignant rhabdoid tumor and Wilms tumor (Fig. 1). Notably, the median *DLL3* expression level in neuroblastomas was higher than in SCLC (*n* = 79), albeit with a broader range of expression.

### DLL3 Protein Expression

**FIGURE 2:**
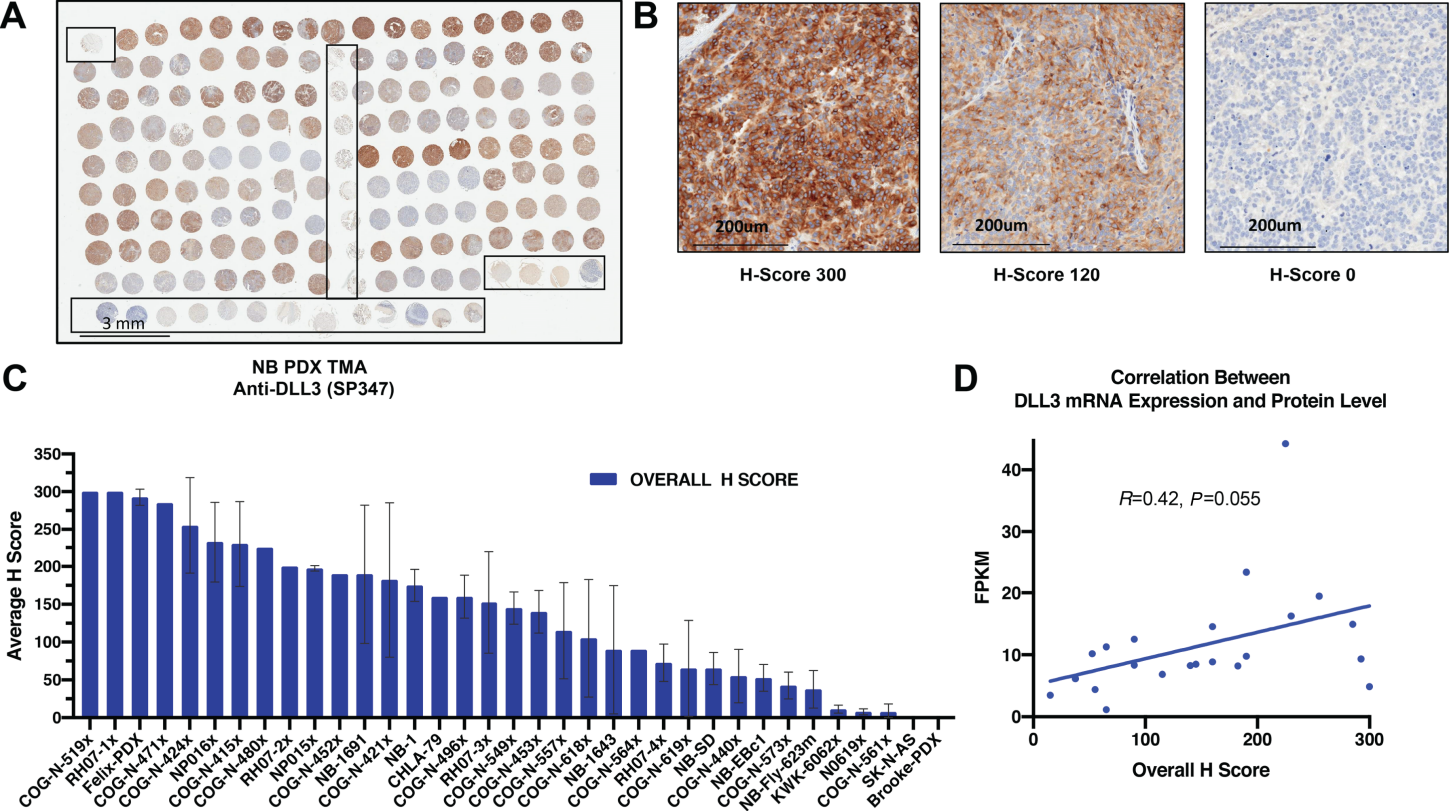
DLL3 is widely expressed in neuroblastoma PDX models. **A,** DLL3 staining of neuroblastoma PDX microarray (TMA) consisting of 35 different neuroblastoma PDX models with duplicate tumor samples and two separate cores per each tumor sample (scale bar = 3 mm). **B,** Representative images of low, medium, and high expressing PDX tumors and their overall H score values (scale bar = 200 μm). **C,** Bar plot of neuroblastoma PDX overall average IHC H scores (highest to lowest). The neuroblastoma PDX TMA was scored for intensity from 0 to 3 (weak to strong) and percentage of staining. A modified score was calculated as intensity multiplied by percentage of positively stained cells. All cores for each of the two tumors per PDX model were averaged for the final score. Data shown as mean ± SEM (*n* = 2). **D,** Correlation plot between DLL3 mRNA expression and protein overall H score.

We next optimized an IHC assay using a commercially available anti-DLL3 mAb (Ventana, SP347 clone) to evaluate DLL3 expression in a panel of neuroblastoma PDX models (Fig. 2A–C). This array contains 140 cores from 35 models with two tumors from each model and two cores from each tumor. The majority of the cores showed completely undifferentiated neuroblastic histology, with 49 of 140 cores (35%) showing some neuropil (generally minimal), and two of these cores (from the same tumor) showed rare ganglion cell differentiation.

**FIGURE 3:**
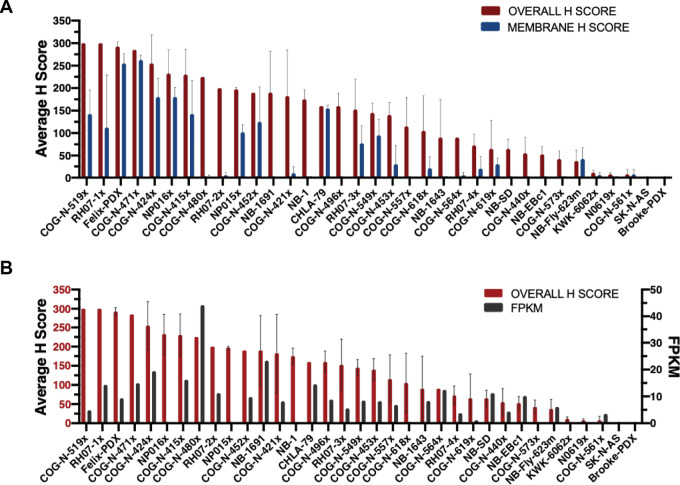
DLL3 shows both membranous and nonmembranous expression in PDX models. **A,** In comparison with the overall H-score, the membrane-specific H-score in any single tumor is generally lower, reflecting the variably prominent subset of cells showing discrete membranous localization. **B,** Correlation between DLL3 mRNA expression and overall H-score in each PDX model.

There was a wide range of DLL3 staining across the 35 models ranging from intense membranous staining to no staining at all (Fig. 2C). In some tumors, there was clear membranous staining, often with cytoplasmic staining as well, others showed cytoplasmic staining only, and in others there was paranuclear accumulation of the signal (Supplementary Fig. S1). Mixtures of patterns were also identified. In most cases with membranous staining or paranuclear staining, a subset of cells (sometimes only a small minority) showed clear membranous (Supplementary Fig. S1A), or paranuclear accentuation (Supplementary Fig. S1C), while a larger number of cells had diffuse (usually weaker) cytoplasmic staining (Supplementary Fig. S1B), resulting in a lower membranous specific score. Some degree of 2+ or 3+ (i.e., greater than equivocal) staining was seen in 116 of 140 cores (83%) or 30 of 35 (86%) of PDX models. Staining was overall concordant between the two cores, with occasionally one degree of difference in intensity between cores. RNA-sequencing data from 21 of the 35 PDX models included on neuroblastoma PDX TMA showed weak correlation with the overall staining IHC H scores (Fig. 2D and Fig. 3).

A human neuroblastoma tumor array with 33 specimens from initial diagnosis, 25 from postchemotherapy local resections, and five from relapsed and/or progressive disease (and one from unknown timing) was also stained. A total of 125 cores were evaluable for DLL3 staining from 64 unique cases (Supplementary Fig. S2). In general, there was much weaker DLL3 staining on this array compared with the PDX array and the robustly expressed cell surface protein NCAM1 (CD56; Supplementary Fig. S3). Fifty cores (40%) from 28 cases (44%) contained at least some cells with staining, but with a very wide range from 1% to 100% of cells (Supplementary Fig. S2 and S3). Only two cases showed an H-score > 100.

The normal human childhood TMA (Supplementary Fig. S4) showed no clear membranous DLL3 staining in any normal tissue (Supplementary Fig. S2). There was weak cytoplasmic staining of placental syncytiotrophoblasts as well as the epithelium in the appendix, ileocecum, fallopian tube, bladder, and gallbladder with supranuclear granular accentuation in the gallbladder. Weak cytoplasmic staining was also seen in a subset of bone marrow mononuclear cells, and in a subset of adrenal cortical cells with a somewhat granular pattern. Moderate granular cytoplasmic staining was present in a subset of neurons and in a subset of gastric glandular epithelial cells. Strong paranuclear cytoplasmic staining was present in a subset of thyroid follicular cells.

### Rova-T Exhibits Antitumor Activity in PDX Models of Neuroblastoma

**FIGURE 4:**
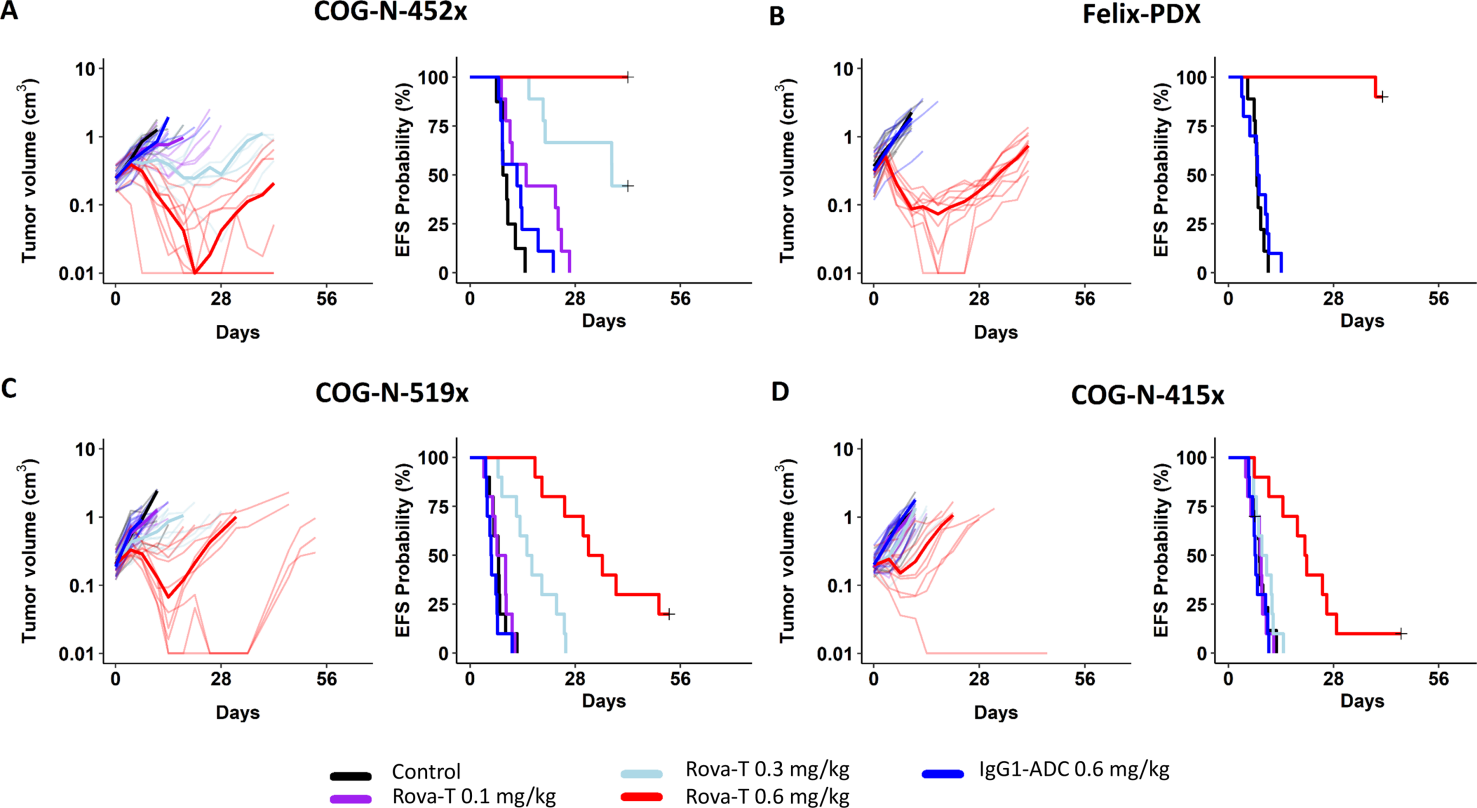
Rova-T induces antitumor activity in DLL3-expressing neuroblastoma PDX models. The *in vivo* efficacy of Rova-T was evaluated in female CB17 SCID mice bearing COG-N-452x (**A**) and Felix PDX (**B**) and COG-N-519x **(C)** and COG-N-415x **(D)**. Upon enrollment, mice (*n* = 8–10 per group) were intraperitoneally injected with 5% glucose water (vehicle control), IgG1-ADC at 0.6 mg/kg or Rova-T at 0.1, 0.3, or 0.6 mg/kg. Differences in EFS between experimental groups (e.g., treated vs. controls) are tested using the Peto and Peto modification of the Gehan–Wilcoxon test (*α* = 0.05, two-sided alternative) and plotted as Kaplan–Meier EFS curves.

**FIGURE 5:**
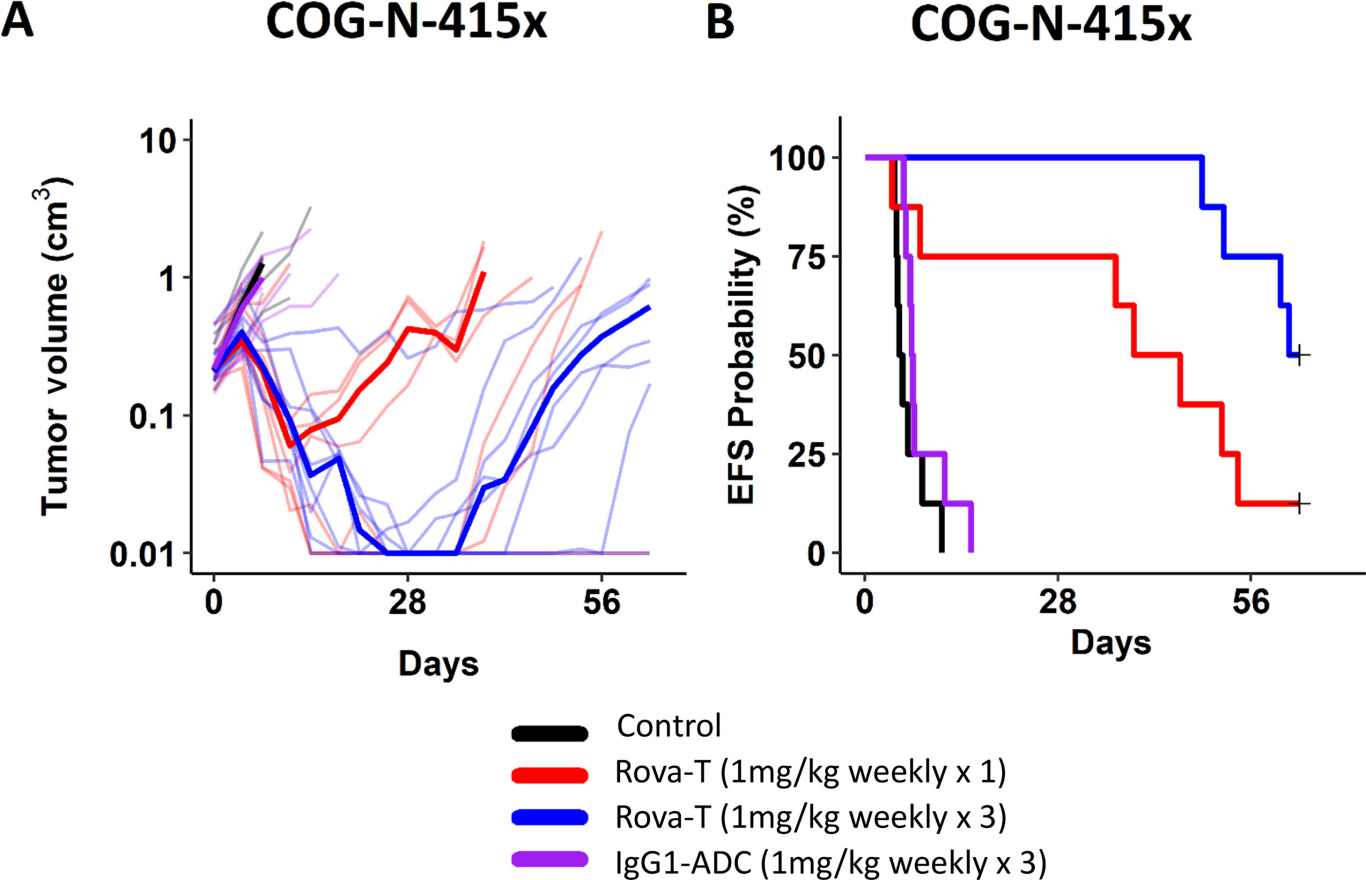
Rova-T increments of dose and schedule induced more sustained antitumor activity in the COG-N-415x neuroblastoma model. Mice bearing the COG-N-415x model received one or three injections of Rova-T or IgG1-ADC (1 mg/kg) every 7 days. **A,** Tumor volume. **B,** EFS probabilities.

We first tested the efficacy of Rova-T in four neuroblastoma PDX models using a conventional design of 8–10 mice per arm. In the PDX array, all four of these models had robust DLL3 expression in the majority of tumor cells including some degree of clearly membranous staining (Supplementary Fig. S5). There was a clear dose–response effect, and all models showed some or significant evidence of antitumor activity at a single 0.6 mg/kg dose (i.e., one injection) of Rova-T (Table 1; Fig. 4; Supplementary Fig. S6). Doses of 0.3 and 0.6 mg/kg induced statistically significant increases in EFS, except for the COG-N-415x dosed at 0.3 mg/kg (Table 1; Fig. 4). We next compared a single 1 mg/kg dose to 1 mg/kg weekly ×3 in mice bearing the COG-N-415x PDX, showing a maintained complete response for 7 weeks with the weekly ×3 treatment schedule, with slow resumption of tumor growth thereafter (Fig. 5; Table 1).

**TABLE 1:** Summary of statistical analysis of *in vivo* studies

Models	Rova-T dose	*N*	Na	KM Med (days)	EFS T − C (days)	EFS T/C	*P*	minRTV Mean SD (cm^3^)*	Median response	FPKM	Modified H score
**COG-N-452x**	0.6 mg/kg	9	9	>42.0	>32.8	>4.55	*P* < 0.001	0.087 ± 0.121	CR	9.8	190.0
	0.3 mg/kg	9	9	37.8	28.5	4.09	*P* < 0.001	1.014 ± 0.626	PD2		
**Felix-PDX**	0.6 mg/kg	10	10	>41	>33.5	>5.45	*P* < 0.001	0.145 ± 0.121	PR	9.3	292.5
**COG-N-519x**	0.6 mg/kg	10	10	33.4	25.9	4.46	*P* < 0.001	0.696 ± 0.833	SD	4.9	300.0
	0.3 mg/kg	10	10	15.8	8.3	2.11	*P* < 0.001	2.003 ± 0.697	PD2		
**COG-N-415x**	0.6 mg/kg	10	10	20.6	12.3	2.49	*P* = 0.001	0.973 ± 0.840	PD2	16.2	230.0
	0.3 mg/kg	10	10	9.5	1.2	1.15	*P* = 0.370	1.619 ± 0.756	PD1		
**COG-N-415x**	1 mg/kg	8	8	42.3	37.2	8.19	*P* = 0.015	1.027 ± 1.685	PR	16.2	230.0
	1 mg/kg (x3)	8	8	61.5	56.3	11.89	*P* < 0.001	0.159 ± 0.430	CR		
**COG-N-424x**	0.6 mg/kg	2	2	22.7	18.7	5.64	*P* = 0.102	0.747 ± 0.837	SD	19.5	255.0
**SH-SY5Y**	0.6 mg/kg	2	2	>36	>31.5	>8.08	*P* = 0.157	0.363 ± 0.514	SD	N/A	N/A
**COG-N-421x**	0.6 mg/kg	2	2	>20	>13.3	>2.97	*P* = 0.102	0.468 ± 0.094	SD	8.2	182.5
	0.1 mg/kg	2	2	8	1.3	1.19	*P* = 1.000	2.825 ± 0.676	PD1		
**KWK-6062x**	0.6 mg/kg	2	2	38.4	15.1	1.65	*P* = 0.102	0.581 ± 0.138	PD2	N/A	11.3
	0.1 mg/kg	2	2	25.5	2.2	1.09	*P* = 0.102	0.850 ± 0.224	PD1		
**SK-N-AS**	0.6 mg/kg	2	2	11.6	−2.1	0.85	*P* = 1.000	0.943 ± 0.781	PD2	N/A	0.0
	0.1 mg/kg	2	2	14.3	0.6	1.04	*P* = 1.000	1.298 ± 0.609	PD1		
**NB-FLY-623m**	0.6 mg/kg	2	2	>28	>13	>1.87	*P* = 0.102	0.804 ± 0.286	PD1	6.1	37.5
	0.1 mg/kg	2	2	18.2	3.2	1.21	*P* = 0.450	1.225 ± 0.420	PD1		

Abbreviations: CR, complete response, disappearance of measurable tumor mass during study period; EFS T − C, difference in median time-to-event (days) between T and C groups; EFS T/C, ratio of median time-to-event (days) between T and C groups; KM Med, Kaplan–Meier estimate of median time-to-event (days); Median response, median response evaluation.; N, total number of mice entering experiment; Na, number of mice in analysis; PD, progressive disease, <50% tumor regression throughout study and >25% tumor growth at end of study; PD1, when PD (progressive disease) and the mouse's time to event ≤200% the Kaplan–Meier median time-to-event in control group; PD2, when PD but, additionally, time-to-event is > 200% of the Kaplan-Meier median time-to-event in control group; PR, partial response, ≥50% tumor regression at any point during study but measurable tumor throughout study period.

* minRTV Mean values shown with standard deviations.

Next, we sought to further understand biomarkers of antitumor activity by studying six additional PDX and CDX models with varying levels of DLL3 expression using an *n* = 2 design (10), with one single dose of vehicle or Rova-T at 0.1 or 0.6 mg/kg. No objective responses were observed and there was SD in three models at 0.6 mg/kg (Table 1). There were no overt signs of toxicity and no treatment-related mortality (Supplementary Fig. S7).

## Discussion

Improved clinical outcomes for patients with high-risk pediatric cancers, especially those with solid tumors, have been hampered by aggressive and nonspecific multimodal therapy such as empiric high-dose chemotherapy. There is an urgent need to identify new drug targets that can be harnessed for the development of more rationale therapies. The Notch pathway is a highly conserved cell-cell signaling pathway involved in a variety of cellular processes and controls fate decisions in developing organs (11–13). Here, we show that the Notch ligand DLL3 is robustly expressed in most high-risk neuroblastoma preclinical models, but much less so in the patient tumor samples. We hypothesize that the predominant adrenergic cell type of neuroblastomas must maintain suppression of Notch signaling, and that PDXs are enriched for adrenergic cell type and thus DLL3 overexpression (14–16). The low protein expression noted in our human tumor array must be viewed in the context that many of the samples were derived from definitive surgery after chemotherapy, and thus enriched for a more mesenchymal cell type (17). Thus, any DLL3-targeting clinical trial should strongly consider real-time assessment of DLL3 protein expression at study entry and explore a minimum expression level required for antitumor efficacy.

Preclinical studies support the development of novel therapies that target DLL3 in SCLC and possibly other neuroendocrine tumors, and have demonstrated that targeting of DLL3 using an ADC approach inhibits tumor growth (5, 18–20). The efficacy seen with Rova-T against a panel of neuroblastoma PDXs suggests that DLL3 is a potential candidate therapeutic target in this disease. Rova-T was evaluated in a pivotal phase II study in patients with DLL3-expressing SCLC (defined by IHC). There was no difference in overall survival between patients high or low DLL3 expression, and results demonstrated modest clinical activity with associated toxicities (21). All patients received 0.3 mg/kg Rova-T intravenously infused over 30 minutes once every 6 weeks for two cycles as the initial treatment. Ultimately, a phase III placebo-controlled trial evaluating Rova-T as a first-line maintenance treatment therapy for advanced SCLC demonstrated no survival benefit and Research and Development of this drug was hence discontinued. A recent trial combining Rova-T with nivolumab plus or minus ipilimumab in patients with relapsed/refractory SCLC showed an objective response rate of 30% but unfortunately the combination was not well tolerated (22). Despite the cessation of trials with Rova-T as an ADC approach using a PBD dimer to target DLL3, this remains a conceivable target in neuroblastoma and other neuroendocrine tumors due to its relatively restricted normal expression and moderate expression in preclinical PDX models. However, in light of the marginal expression on the cell surface of human neuroblastoma tumors, this target should be contemplated with caution for immunotherapeutic approaches that exploit not only antigen specificity but also and receptor density.

DLL3-targeted Bispecific T-cell Engager (BiTE) molecules and chimeric antigen receptors (CAR) T cells are in development to promote the tumor-suppressive functions of DLL3 and to avoid oncogenicity. These include Amgen's 757 Anti-DLL3 x CD3 BiTE antibody (23) and AMG 119 CAR, as well as Boehringer Ingelheim's bispecific DLL3/CD3 IgG-like T-cell engaging antibody (24). In addition, we should not rule out the possibility of other DLL3-ADC approaches using different payloads. Clinical trials suggest that a better understanding of the mechanistic basis of ADC activity as well as exploration and modification of dose and schedule of DLL3-targeted ADC are necessary to reduce toxicity and improve efficacy. Along these lines, recent studies indicate that in addition to intracellular release of the cytotoxic payload, ADCs can concomitantly suppress infiltrating lymphocytes that overall limits their efficacy (25).

Collectively, these data provide a foundation for vigilance about future consideration of clinical development of DLL3-targeted immunotherapeutic agents for neuroblastoma and other potentially other DLL3-expressing neuroendocrine tumors. Any neuroblastoma clinical trial must consider the heterogeneity and plasticity of DLL3 expression on Notch pathway activity in this disease.

## Supplementary Material

Figures S1-7 Supplementary Figures 1-7 with legends included
