# Supplementary material for: Evaluation of the DLL3-targeting Antibody–Drug Conjugate Rovalpituzumab Tesirine in Preclinical Models of Neuroblastoma
Source: Cancer Res Commun. 2022 Jul 11;2(7):616–23. doi: 10.1158/2767-9764.CRC-22-0137 (PMC9648412; doi:10.1158/2767-9764.CRC-22-0137)
Supplement: Figures S1-7 — Supplementary Figures 1-7 with legends included [file crc-22-0137-s01.pptx]

## Slide 1
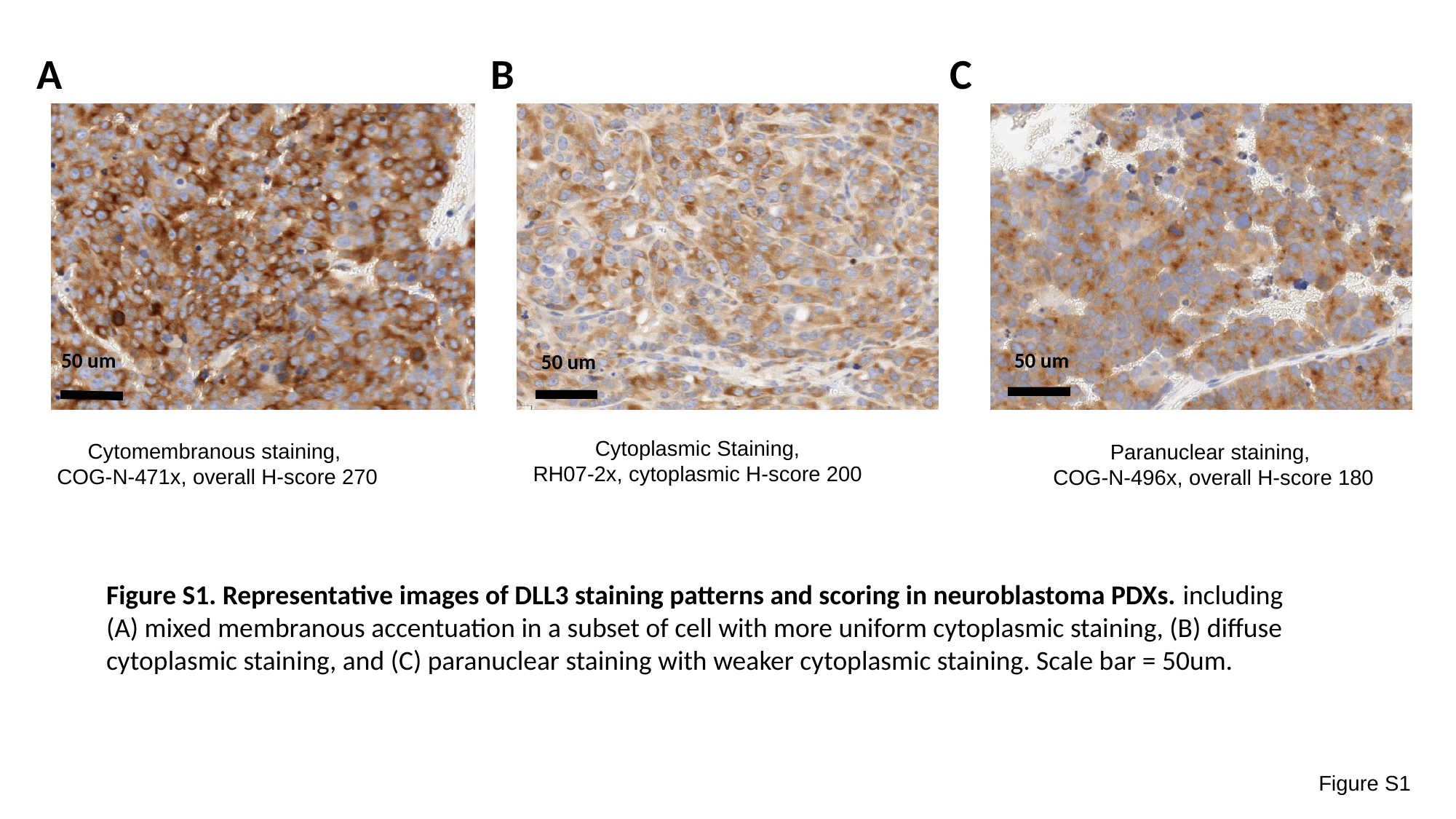

A
B
C
50 um
50 um
50 um
Cytoplasmic Staining,
RH07-2x, cytoplasmic H-score 200
Cytomembranous staining,
COG-N-471x, overall H-score 270
Paranuclear staining,
COG-N-496x, overall H-score 180
Figure S1. Representative images of DLL3 staining patterns and scoring in neuroblastoma PDXs. including (A) mixed membranous accentuation in a subset of cell with more uniform cytoplasmic staining, (B) diffuse cytoplasmic staining, and (C) paranuclear staining with weaker cytoplasmic staining. Scale bar = 50um.
Figure S1

## Slide 2
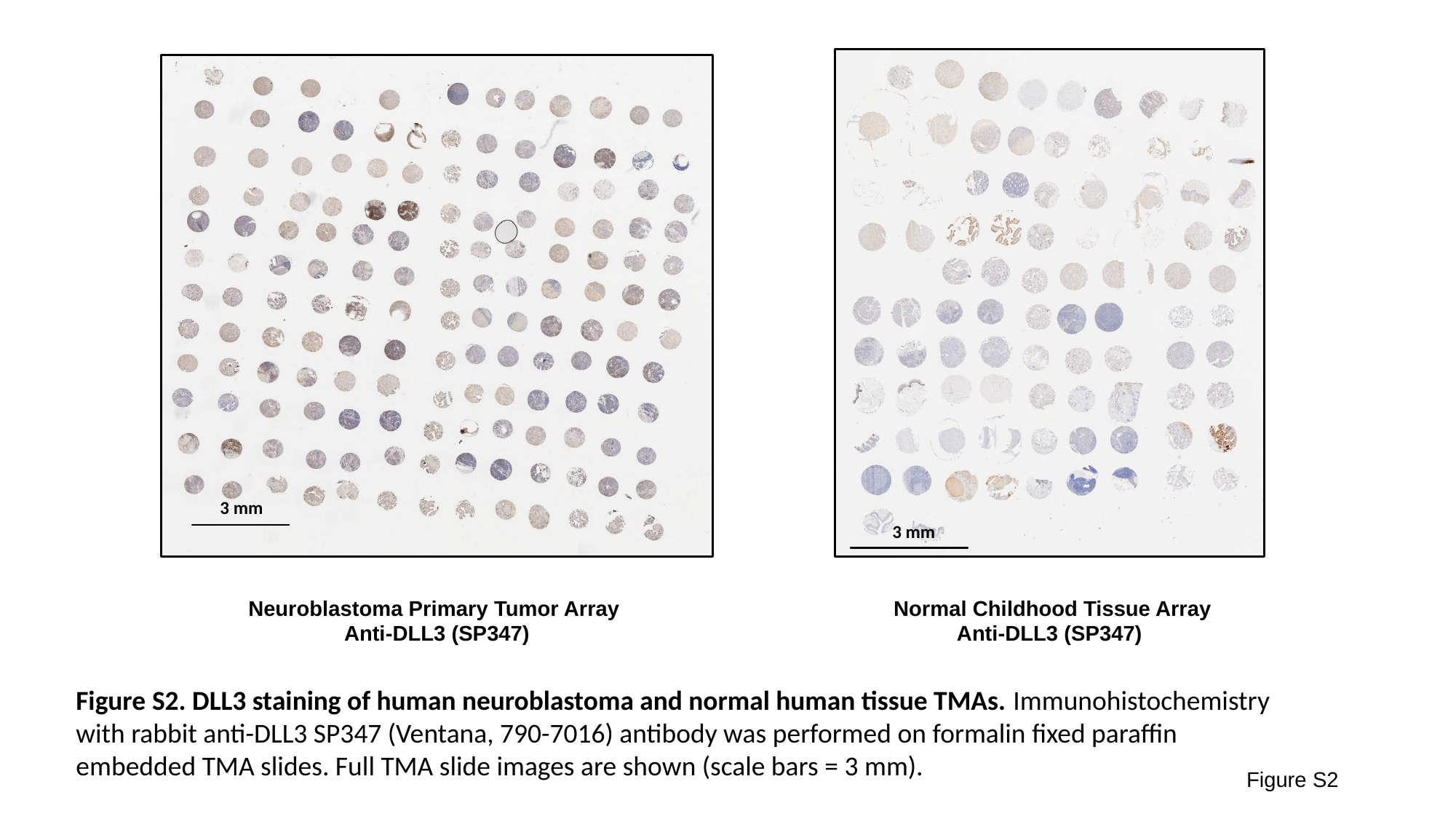

Normal Childhood Tissue Array
Anti-DLL3 (SP347)
3 mm
Neuroblastoma Primary Tumor Array
Anti-DLL3 (SP347)
3 mm
Figure S2. DLL3 staining of human neuroblastoma and normal human tissue TMAs. Immunohistochemistry with rabbit anti-DLL3 SP347 (Ventana, 790-7016) antibody was performed on formalin fixed paraffin embedded TMA slides. Full TMA slide images are shown (scale bars = 3 mm).
Figure S2

## Slide 3
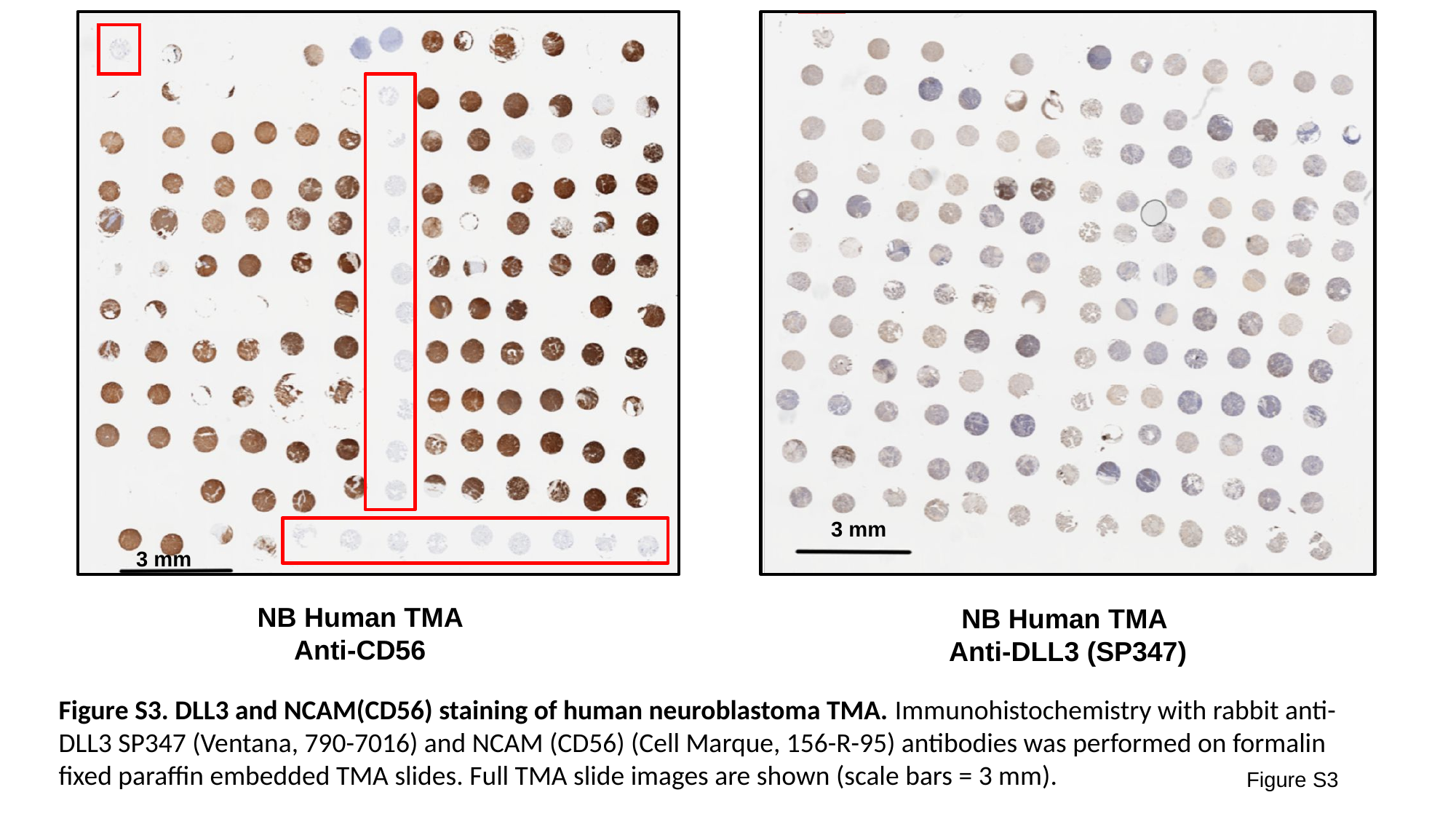

3 mm
3 mm
NB Human TMA
Anti-CD56
NB Human TMA
Anti-DLL3 (SP347)
Figure S3. DLL3 and NCAM(CD56) staining of human neuroblastoma TMA. Immunohistochemistry with rabbit anti-DLL3 SP347 (Ventana, 790-7016) and NCAM (CD56) (Cell Marque, 156-R-95) antibodies was performed on formalin fixed paraffin embedded TMA slides. Full TMA slide images are shown (scale bars = 3 mm).
Figure S3

## Slide 4
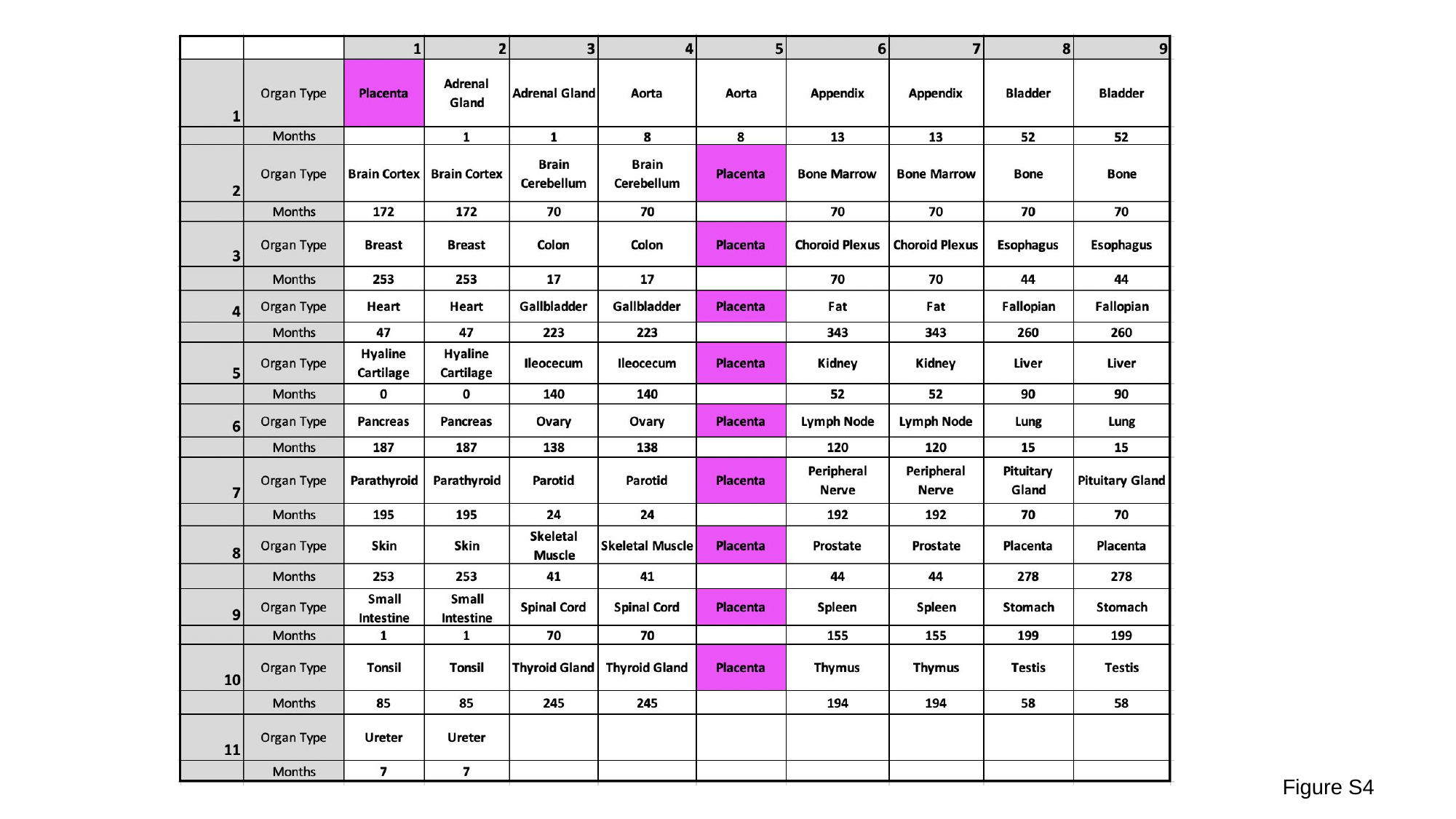

Figure S4

## Slide 5
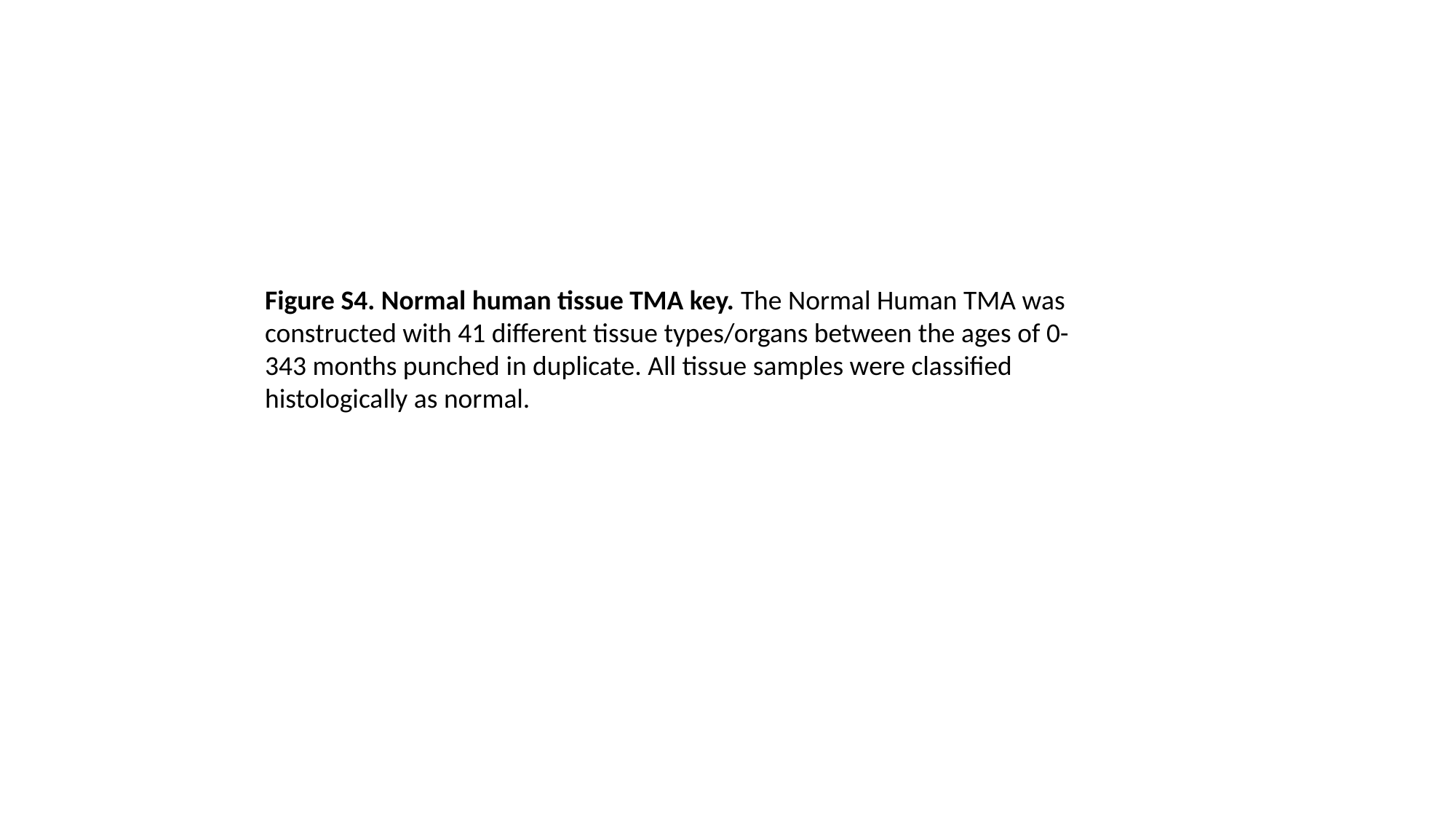

Figure S4. Normal human tissue TMA key. The Normal Human TMA was constructed with 41 different tissue types/organs between the ages of 0-343 months punched in duplicate. All tissue samples were classified histologically as normal.

## Slide 6
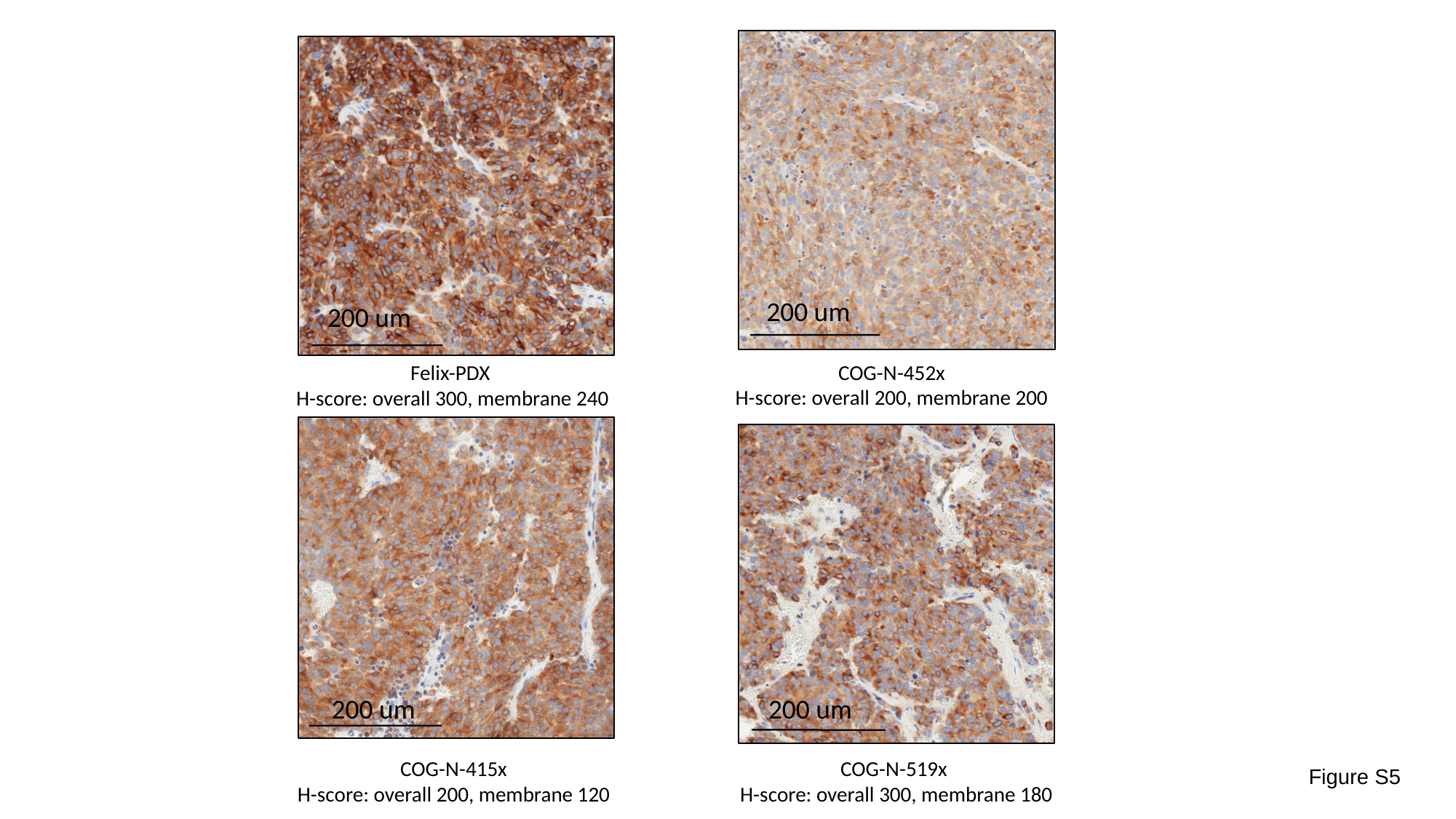

200 um
200 um
COG-N-452x
H-score: overall 200, membrane 200
Felix-PDX
H-score: overall 300, membrane 240
200 um
200 um
COG-N-415x
H-score: overall 200, membrane 120
COG-N-519x
H-score: overall 300, membrane 180
Figure S5

## Slide 7
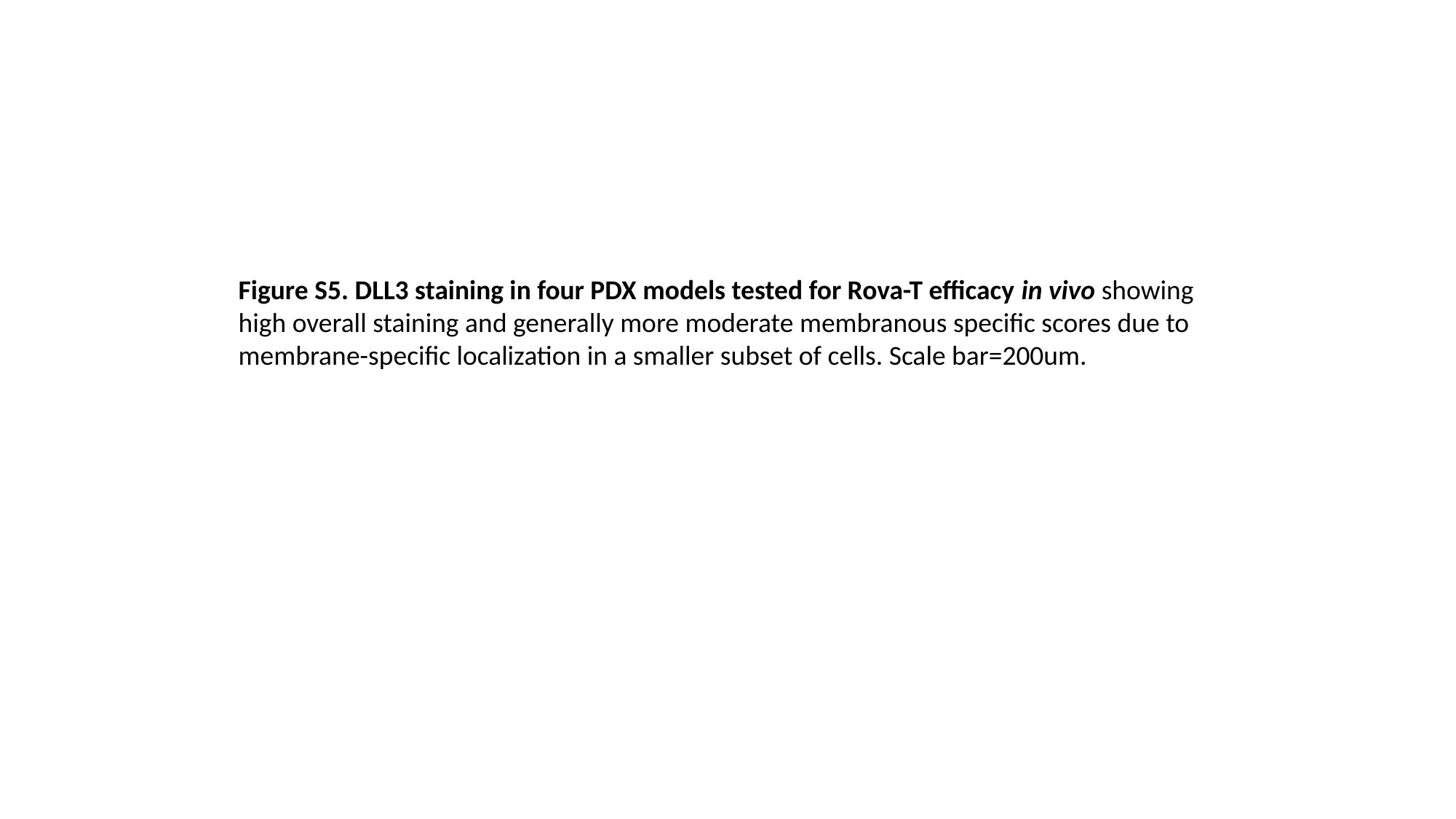

Figure S5. DLL3 staining in four PDX models tested for Rova-T efficacy in vivo showing high overall staining and generally more moderate membranous specific scores due to membrane-specific localization in a smaller subset of cells. Scale bar=200um.

## Slide 8
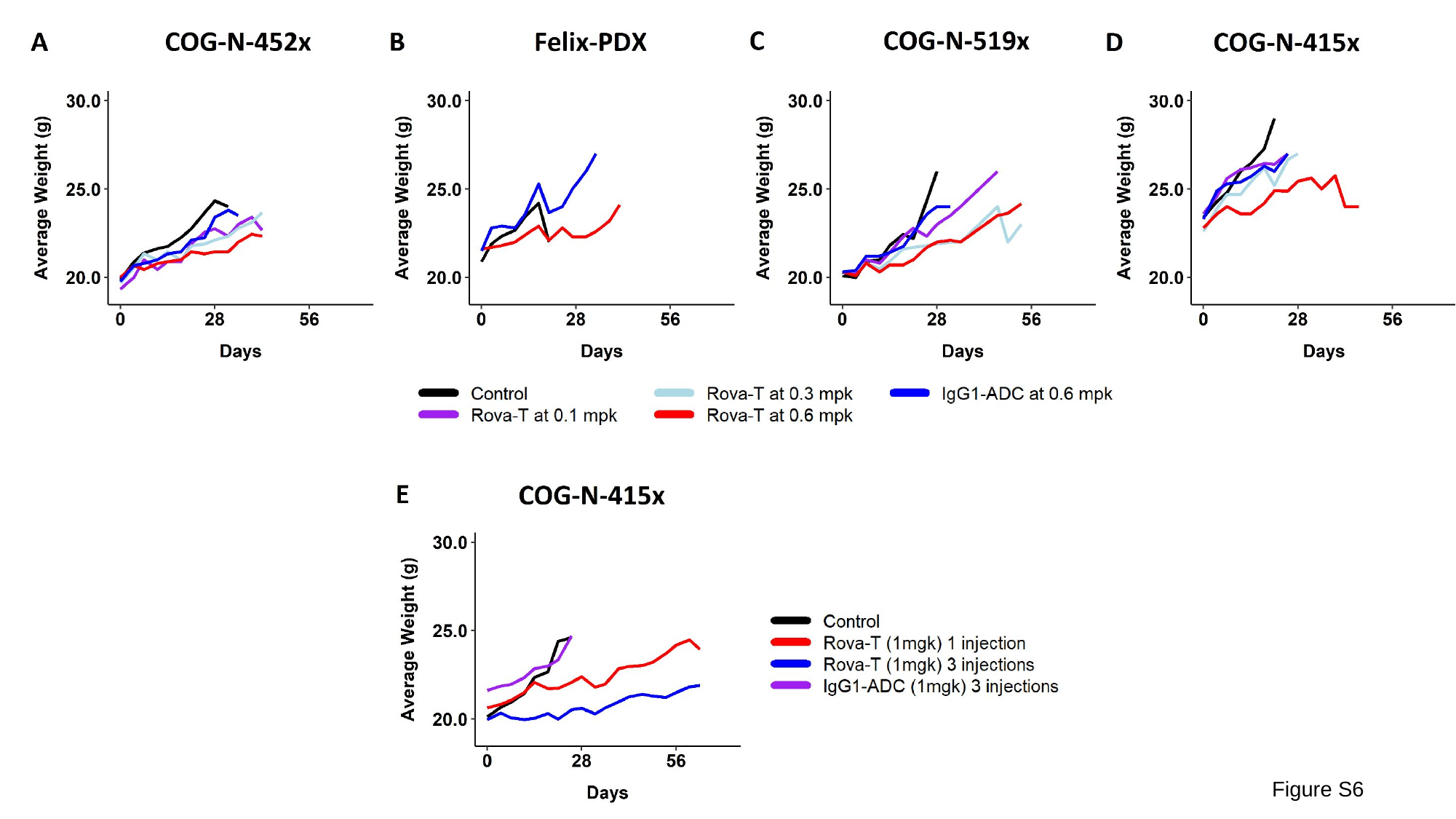

Figure S6

## Slide 9
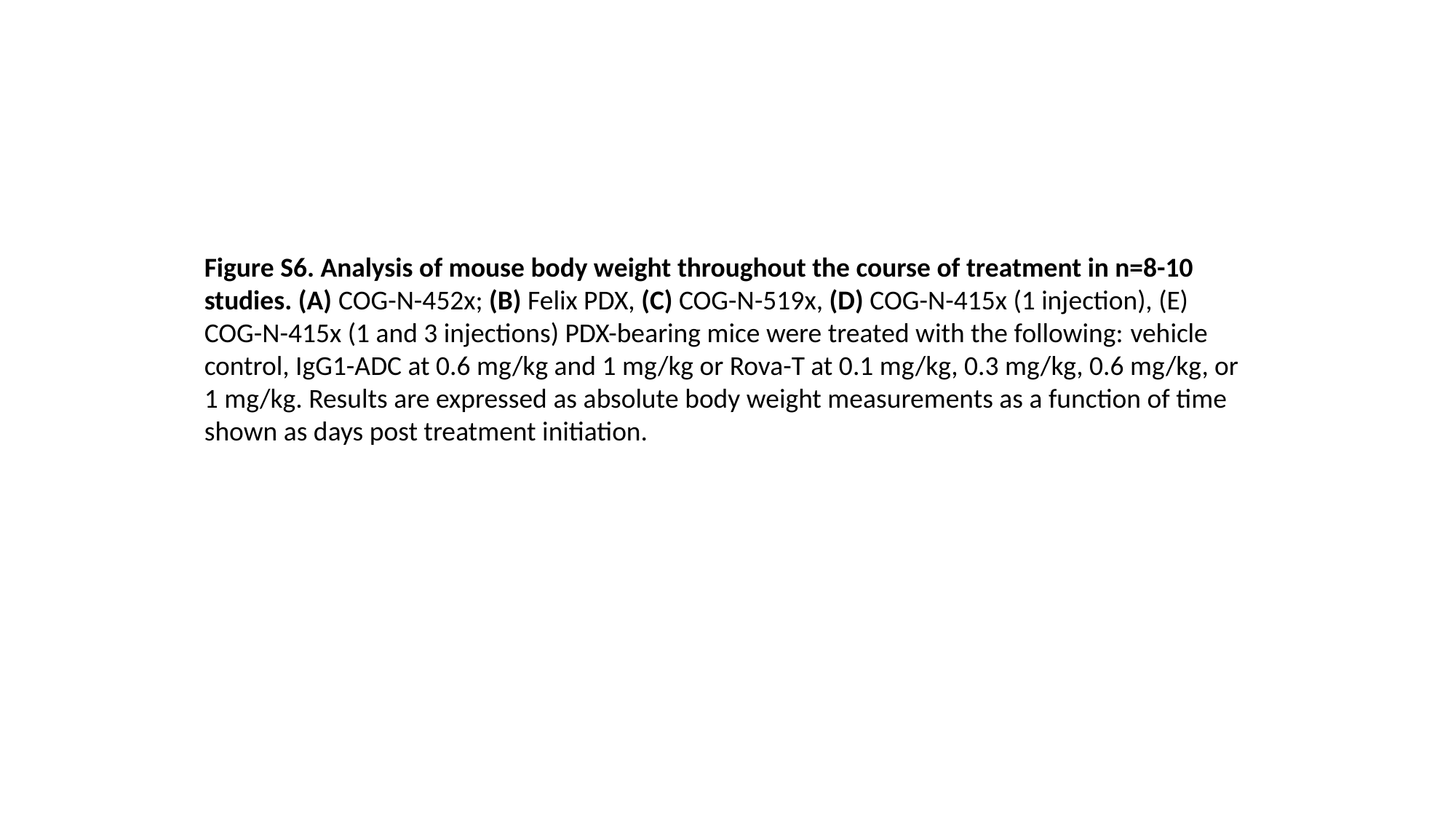

Figure S6. Analysis of mouse body weight throughout the course of treatment in n=8-10 studies. (A) COG-N-452x; (B) Felix PDX, (C) COG-N-519x, (D) COG-N-415x (1 injection), (E) COG-N-415x (1 and 3 injections) PDX-bearing mice were treated with the following: vehicle control, IgG1-ADC at 0.6 mg/kg and 1 mg/kg or Rova-T at 0.1 mg/kg, 0.3 mg/kg, 0.6 mg/kg, or 1 mg/kg. Results are expressed as absolute body weight measurements as a function of time shown as days post treatment initiation.

## Slide 10
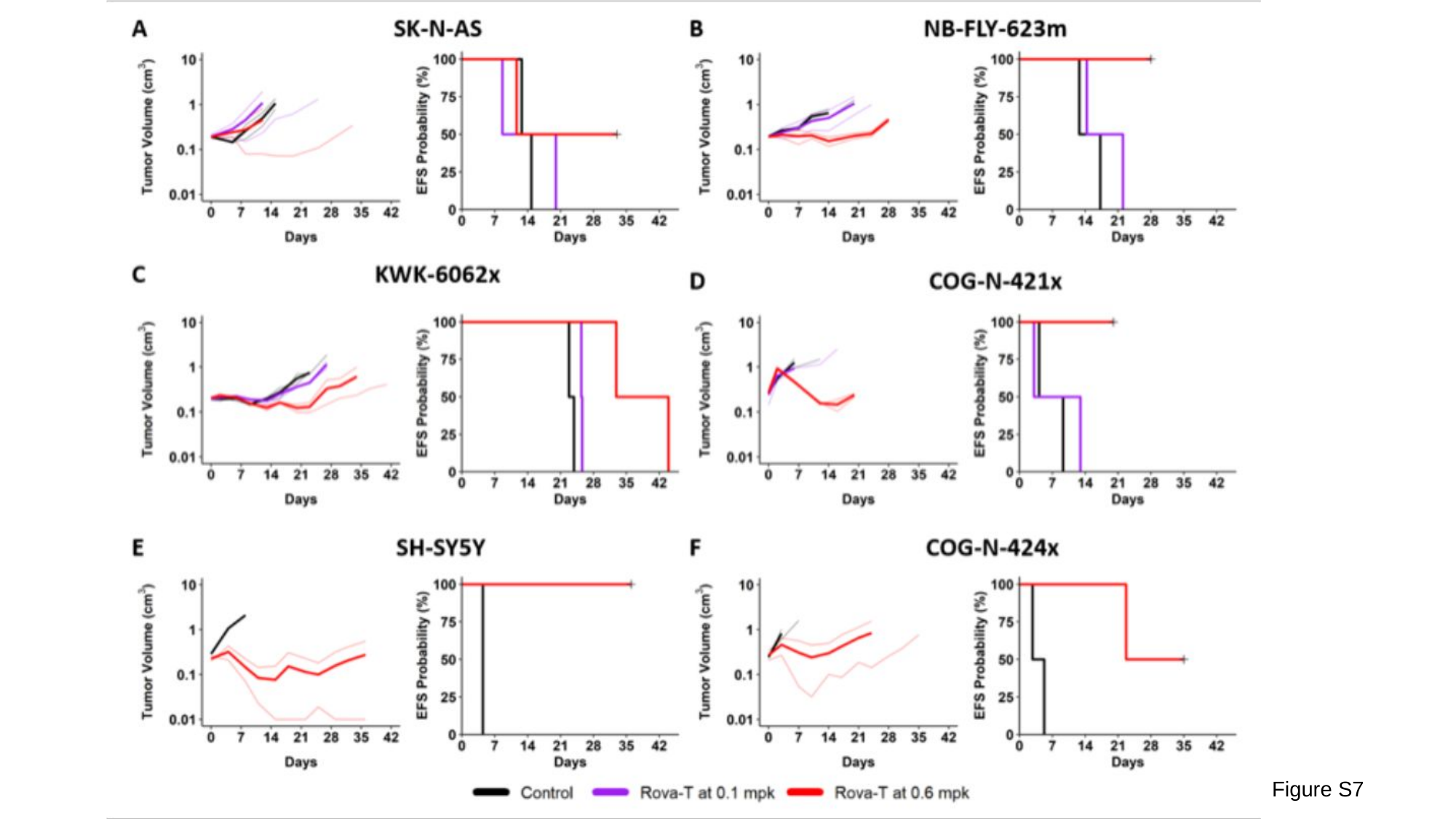

Figure S7

## Slide 11
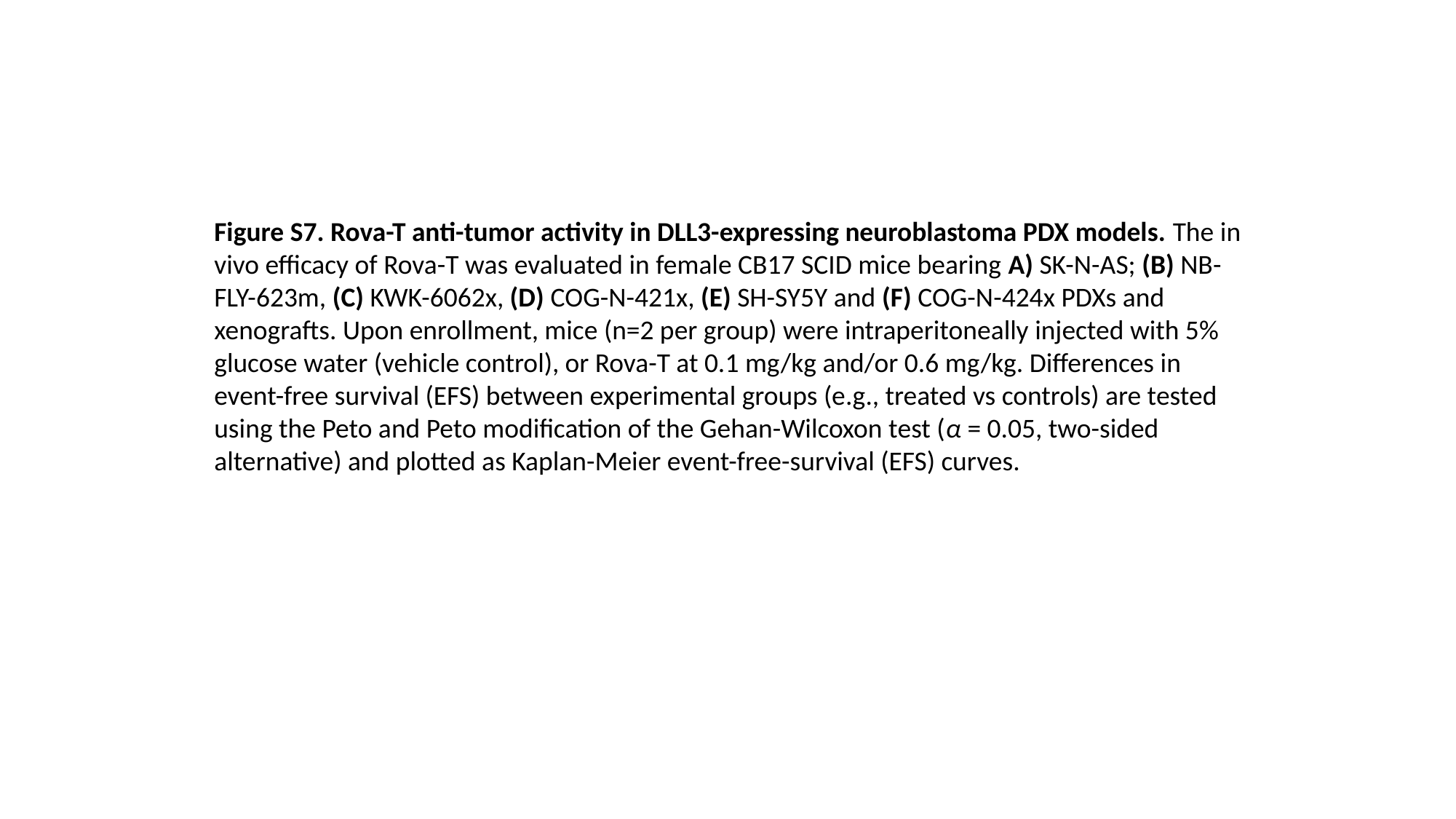

Figure S7. Rova-T anti-tumor activity in DLL3-expressing neuroblastoma PDX models. The in vivo efficacy of Rova-T was evaluated in female CB17 SCID mice bearing A) SK-N-AS; (B) NB-FLY-623m, (C) KWK-6062x, (D) COG-N-421x, (E) SH-SY5Y and (F) COG-N-424x PDXs and xenografts. Upon enrollment, mice (n=2 per group) were intraperitoneally injected with 5% glucose water (vehicle control), or Rova-T at 0.1 mg/kg and/or 0.6 mg/kg. Differences in event-free survival (EFS) between experimental groups (e.g., treated vs controls) are tested using the Peto and Peto modification of the Gehan-Wilcoxon test (α = 0.05, two-sided alternative) and plotted as Kaplan-Meier event-free-survival (EFS) curves.
